# Risk Stratification, Measurable Residual Disease, and Outcomes of AML Patients with a Trisomy 8 Undergoing Allogeneic Hematopoietic Stem Cell Transplantation

**DOI:** 10.3390/cancers13225679

**Published:** 2021-11-13

**Authors:** Donata Backhaus, Madlen Jentzsch, Lara Bischof, Dominic Brauer, Christina Wilhelm, Julia Schulz, Georg-Nikolaus Franke, Wolfram Pönisch, Vladan Vucinic, Uwe Platzbecker, Sebastian Schwind

**Affiliations:** Medical Clinic and Policlinic 1, Hematology and Cellular Therapy, Leipzig University Hospital, Liebigstraße 22, Haus 7, 04103 Leipzig, Germany; donata.backhaus@medizin.uni-leipzig.de (D.B.); madlen.jentzsch@medizin.uni-leipzig.de (M.J.); lara.bischof@medizin.uni-leipzig.de (L.B.); dominic.brauer@medizin.uni-leipzig.de (D.B.); christina.wilhelm@medizin.uni-leipzig.de (C.W.); julia.schulz3@medizin.uni-leipzig.de (J.S.); georg-nikolaus.franke@medizin.uni-leipzig.de (G.-N.F.); Wolfram.Poenisch@medizin.uni-leipzig.de (W.P.); vladan.vucinic@medizin.uni-leipzig.de (V.V.); uwe.platzbecker@medizin.uni-leipzig.de (U.P.)

**Keywords:** trisomy 8, AML, allogeneic HSCT, MRD

## Abstract

**Simple Summary:**

Trisomy 8 is the most common numerical chromosome aberration in acute myeloid leukemia (AML). Although this AML type is often consolidated applying allogeneic hematopoietic stem cell transplantations (HSCT), detailed analyses of outcomes after HSCT are lacking. The purpose of this manuscript is to analyze biological and clinical features of patients with this cytogenetic aberration in the context of significant risk factors, including the ELN2017 risk stratification and measurable residual disease markers at HSCT. Our data provides evidence on the clinical disease courses and may aid in informed decisions on treatment and outcome prediction of trisomy 8 AML patients undergoing allogeneic HSCT.

**Abstract:**

Background: For most patients with acute myeloid leukemia (AML) harboring a trisomy 8 an allogeneic hematopoietic stem cell transplantation (HSCT) is a suitable and recommended consolidation therapy. However, comparative outcome analyses between patients with and without trisomy 8 undergoing allogeneic HSCT have not been performed so far. Methods: We retrospectively analyzed clinical features, outcomes, and measurable residual disease (MRD) of 659 AML (12%, *n* = 81, with a trisomy 8) patients subjected to allogeneic HSCT as a consolidation therapy. Results: The presence of a trisomy 8 associated with a trend for higher age at diagnosis, AML of secondary origin, lower white blood cell counts at diagnosis, worse ELN2017 genetic risk, wild-type *NPM1*, and mutated *IDH1/2* and *JAK2*. Outcomes after allogeneic HSCT in the entire cohort did not differ between patients with a sole trisomy 8, trisomy 8 with additional cytogenetic aberrations or without a trisomy 8. A trisomy 8 did not affect outcomes within the three ELN2017 risk groups. In accordance with findings in unselected patient cohorts, persistent MRD at allogeneic HSCT in patients with a trisomy 8 identified individuals with a higher risk of relapse following allogeneic HSCT. Conclusions: Outcomes of trisomy 8 patients after allogeneic HSCT did not compare unfavorably to that of other AML patients following allogeneic HSCT. Rather than the presence or absence of a trisomy 8, additional genetic aberrations and MRD at HSCT define outcome differences and aid in informed treatment decisions.

## 1. Introduction

Trisomy 8 is one of the most common cytogenetic aberrations in myeloid malignancies. In acute myeloid leukemia (AML) an additional chromosome 8 can be found in approximately 10% of the patients [1,2]. Within the last 20 years, most genetic risk classification systems including the Medical Research Council (MRC), as well as the European LeukemiaNet (ELN) in 2010 and 2017 assigned patients with a trisomy 8 without additional cytogenetic (or detectable molecular) aberrations to an intermediate or higher-risk disease [3,4,5]. Matching these, outcomes of patients with a sole trisomy 8 have been reported to be similar to that of patients with a normal karyotype [6,7]. Data also suggested that in patients with a trisomy 8, co-occurring cytogenetic alterations define risk at diagnosis, especially when clearly favorable risk factors (such as a core-binding factor AML) or adverse risk factors (such as abnormal 11q23 or a complex karyotype) are present [7,8,9,10]. 

An allogeneic hematopoietic stem cell transplantation (HSCT) is a suitable post-remission consolidation therapy approach, especially for AML patients with intermediate or high-risk AML [11]. Several retrospective studies already suggested that an allogeneic HSCT might be beneficial for patients harboring a trisomy 8 [8,10,12,13]. In a meta-analysis of the German AML intergroup which analyzed trisomy 8 as either a sole aberration or in combination with one additional genetic variation, patients consolidated by allogeneic HSCT showed improved relapse-free survival (RFS) compared to patients undergoing chemotherapy consolidation or autologous HSCT [8]. This assumption was recently confirmed by the EBMT that reported on 401 AML patients with an isolated trisomy 8 transplanted either with autologous or HLA-matched allogeneic HSCT in first remission. In this study, patients receiving allogeneic HSCT had a lower cumulative incidence of relapse (CIR), and—despite a higher non-relapse mortality (NRM)—longer RFS as well as a trend for longer overall survival (OS) [12]. Additionally, single center as well as registry-based analyzes indicated promising outcomes in larger cohorts of patients with a trisomy 8, further pointing to allogeneic HSCT as an effective consolidation treatment in these patients [10,13].

Within recent years, the importance of risk stratification during disease course by the evaluation of measurable residual disease (MRD) in unselected patient cohorts became increasingly evident, which is also true prior to performing an allogeneic HSCT in morphologic remission [14,15,16,17]. 

Despite the importance of allogeneic HSCT in consolidating intermediate or high-risk trisomy 8 AML patients, outcome analyzes regarding the presence or absence of a trisomy 8 in AML patients undergoing allogeneic HSCT, especially in the context of the MRD status at HSCT, have not been performed so far. The aim of this study was to compare relapse incidences and survival of AML patients with or without a trisomy 8 subjected to allogeneic HSCT in the context of current risk assessment, including the ELN2017 genetic risk stratification, and the MRD status at allogeneic HSCT.

## 2. Material and Methods

### 2.1. Patient Population

A total of 659 AML patients who received an allogeneic HSCT at the University Hospital Leipzig between July 1998 and October 2020 and had karyotype information at diagnosis available were retrospectively included in this analysis. Median age at diagnosis was 59.2 (range 14.3–76.5) years. Patients received HSCT in first complete remission (CR) or CR with incomplete peripheral recovery (68%), second CR/CRi (17%), third CR/CRi (0.3%), or with active disease (20%) after myeloablative (25%), reduced intensity (17%) or non-myeloablative (58%) conditioning. Further patient characteristics are given in Table 1 and Table 2, the Supplementary Material and Supplementary Tables S1 and S2. Written informed consent was obtained in accordance with the Declaration of Helsinki. Median follow up alive after allogeneic HSCT was 3.7 years.

### 2.2. Flow Cytometry, Cytogenetics, and Molecular Markers

For all patients, cytogenetic analyses were performed centrally in our institution using standard banding techniques as previously described [2]. The karyotype description was performed according to the recommendations of the International System for Human Cytogenetic Nomenclature [18]. In patients with pretreatment bone marrow material available, the presence of internal tandem duplication in the *FLT3* gene (*FLT3*-ITD), mutations in the *FLT3* tyrosine kinase domain (*FLT3*-TKD) and in the *NPM1* and *CEBPA* genes and the mutation status of 54 genes included in the TruSight Myeloid Sequencing Panel (Illumina) were evaluated as previously described [19,20,21,22]. Insertion mutations at codon 646 in the gene *ASXL1* were analyzed by Sanger sequencing using a proofreading polymerase as previously reported [23]. Patients were grouped according to the ELN2017 risk classification [4]. Evaluation of the immunophenotype at diagnosis was performed as previously described [21]. 

### 2.3. MRD Status at HSCT

In patients with adequate material available the MRD status at allogeneic HSCT was assessed. In patients with known non-clonal hematopoiesis associated mutations, MRD was assessed by mutation-specific digital droplet PCR assays (based on mutations in *FLT3*-TKD, *IDH1*, *NPM1*, *KIT*, *KRAS*, *NRAS*, *TP53,* and non-canonical *DNMT3A* and *ASXL1* mutations, similarly as previously described [24,25,26]). In patients without known trackable gene mutations, the gene expressions of *BAALC/ABL1*, *MN1/ABL1*, and *WT1/ABL1* were adapted as MRD as previously described using the established cut-offs [27,28,29]. In patients without known trackable gene mutations and without material for gene expression MRD analysis, FISH analyses of at least 100 interphases of the dominant cytogenetic aberration at diagnosis was used as MRD at HSCT. For outcome analyses according to the number of positive MRD markers, all available MRD analyses were added together.

### 2.4. Definition of Clinical Endpoints and Statistical Analyses

All statistical analyses were performed using the R statistical software platform (version 4.0.2) [30]. CIR was calculated from HSCT to morphologic relapse and OS was calculated from HSCT to death from any cause. OS estimates were calculated using the Kaplan-Meier method and groups were compared using the log-rank test. CIR was calculated considering the competing risk NRM using the Fine and Gray model [31]. Associations with baseline clinical, demographic, and molecular features were compared using the Kruskal–Wallis test and Fisher’s exact test for continuous and categorical variables, respectively. Multivariate Analyses are described in the Supplementary Material.

## 3. Results

### 3.1. Incidence of Trisomy 8 in AML Patients Subjected to Allogeneic HSCT

**Figure 1:**
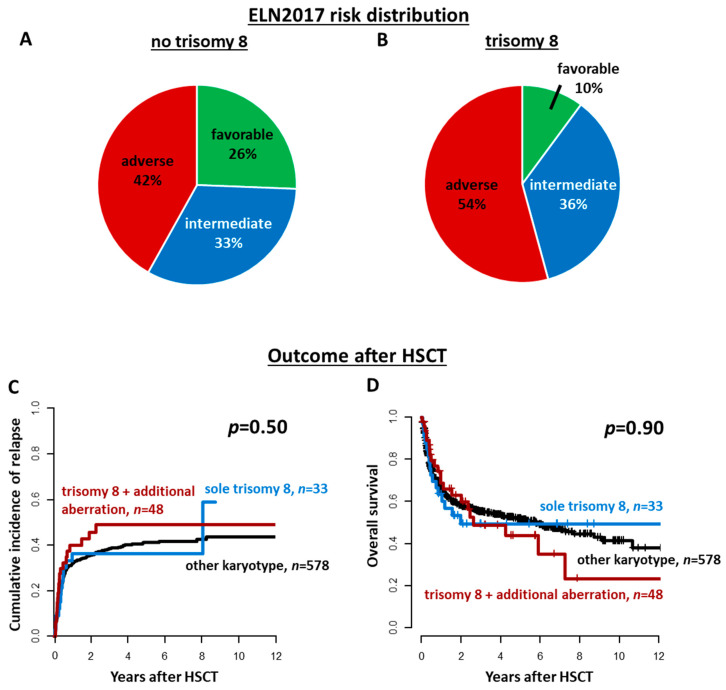
ELN2017 risk distribution in AML patients (**A**) without and (**B**) with a trisomy 8 at diagnosis. Outcomes of AML patients undergoing allogeneic hematopoietic stem cell transplantation (HSCT) according to the presence or absence of a trisomy 8 and additional cytogenetic aberrations (sole trisomy 8 vs. trisomy 8 and additional cytogenetic aberration vs. others, *n* = 659). (**C**) Cumulative incidence of relapse, and (**D**) Overall survival.

Overall, 12% (*n* = 81) of patients harbored a trisomy 8 at diagnosis. Of those, 41% (*n* = 33) had trisomy 8 as a sole cytogenetic aberration, and 59% (*n* = 48) in combination with other cytogenetic aberrations, which are displayed in Supplementary Table S1. According to the MRC cytogenetic classification [5], four trisomy 8 patients had additional favorable risk cytogenetics, 19 patients had additional intermediate risk cytogenetics, and 25 patients had additional adverse risk cytogenetics. Among the latter, 19 patients harbored a complex karyotype. The ELN2017 genetic risk distribution between patients with or without a trisomy 8 as well as between trisomy 8 patients with or without additional cytogenetic aberrations is displayed in Figure 1A,B and Supplementary Figure S1, respectively.

### 3.2. Characteristics of AML Patients Harboring a Trisomy 8

Compared to all others, patients with trisomy 8 were by trend older at diagnosis (*p* = 0.08), and more often had a secondary AML (*p* = 0.04), a lower white blood count (*p* = *0*.01), a higher bone marrow CD3+/CD38− cell burden (*p* = 0.05), and a distinct immunophenotype (Supplementary Table S2) at diagnosis. They had worse ELN2017 risk (*p* = *0*.03), and a higher incidence of a complex karyotype (*p* = 0.03). They also had a lower incidence of *NPM1* mutations (*p* = 0.002), but a higher incidence of *JAK2* (*p* = 0.04) and *IDH1/2* mutations (*p* = 0.05) and had by trend higher *BAALC*/*ABL1* copy numbers at diagnosis (*p* = 0.08). Importantly, patients with a trisomy 8 were more likely to receive an allogeneic HSCT with active disease (*p* = 0.04).

Comparing patients with a sole trisomy 8 to patients with a trisomy 8 and additional cytogenetic aberrations, patients with a sole trisomy 8 were significantly older at AML diagnosis (*p* = 0.04). The incidence of *JAK2* mutations was significantly higher in patients with a sole trisomy 8 (*p* = 0.02), and these patients were also more likely to be *BCOR* mutated (*p* = 0.04) and by trend *TET2* mutated (*p* = 0.06).

### 3.3. Outcomes of AML Patients Harboring a Trisomy 8

**Figure 2:**
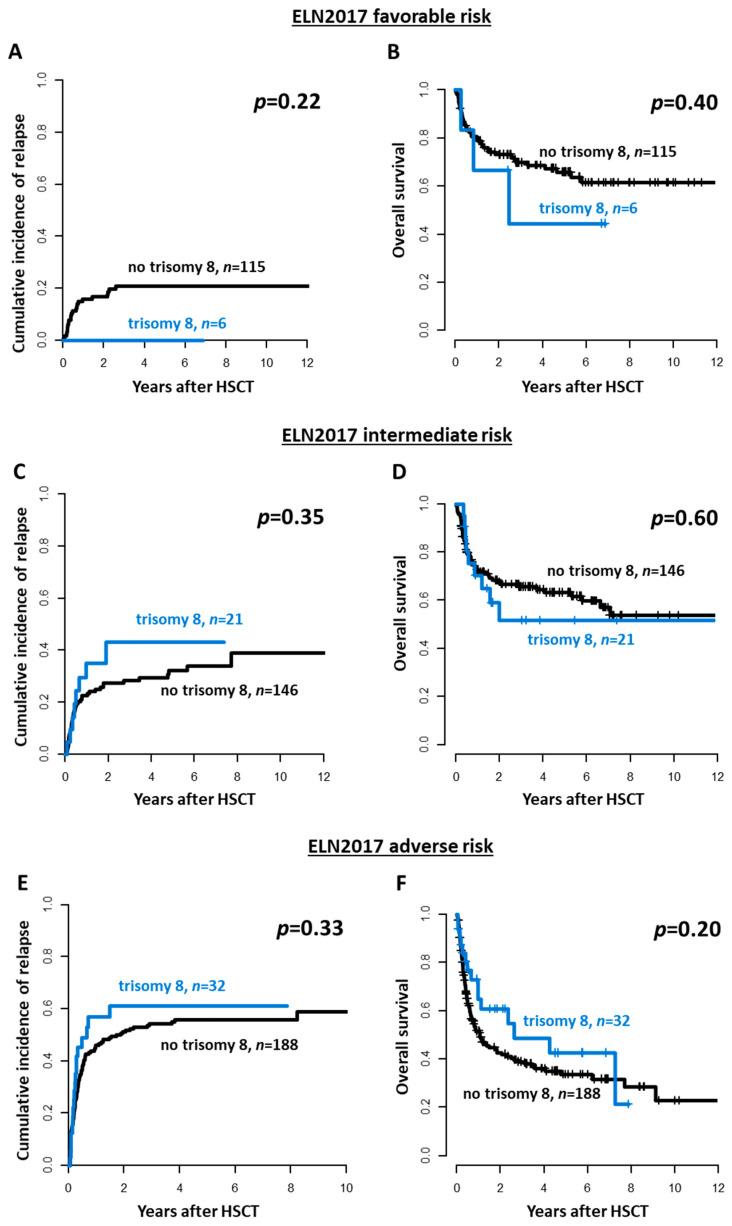
Outcomes of AML patients undergoing allogeneic hematopoietic stem cell transplantation (HSCT) within the three ELN2017 genetic risk groups according to the presence or absence of a trisomy 8 (trisomy 8 vs. others). (**A**) Cumulative incidence of relapse, and (**B**) Overall survival in patients with favorable ELN2017 risk (*n* = 121). (**C**) Cumulative incidence of relapse, and (**D**) Overall survival in patients with intermediate ELN2017 risk (*n* = 167). (**E**) Cumulative incidence of relapse, and (**F**) Overall survival in patients with adverse ELN2017 risk (*n* = 220).

After allogeneic HSCT, patients with a sole trisomy 8 as well as patients with a trisomy 8 and additional cytogenetic aberrations had comparable CIR (*p* = 0.50, Figure 1C) and OS (*p* = 0.90, Figure 1D) as patients without a trisomy 8 at diagnosis. The presence of a trisomy 8 also did not impact CIR or OS in multivariate analyses (Supplementary Table S3). Similar results were obtained when restricting the analysis to patients transplanted in cytomorphologic CR/CRi (CIR, *p* = 0.84 and OS, *p* = 1, Supplementary Figure S2) and first CR/CRi (CIR, *p* = 0.45 and OS, *p* = 0.60, Supplementary Figure S3). Additionally, within the three ELN2017 risk groups, outcomes did not differ significantly between patients with or without a trisomy 8 at diagnosis (ELN2017 favorable: CIR *p* = 0.22 and OS *p* = 0.40, ELN2017 intermediate: CIR *p* = 0.35 and OS *p* = 0.60, ELN2017 adverse: CIR *p* = 0.33 and OS *p* = 0.20, Figure 2).

### 3.4. MRD at HSCT in AML Patients Harboring a Trisomy 8

**Figure 3:**
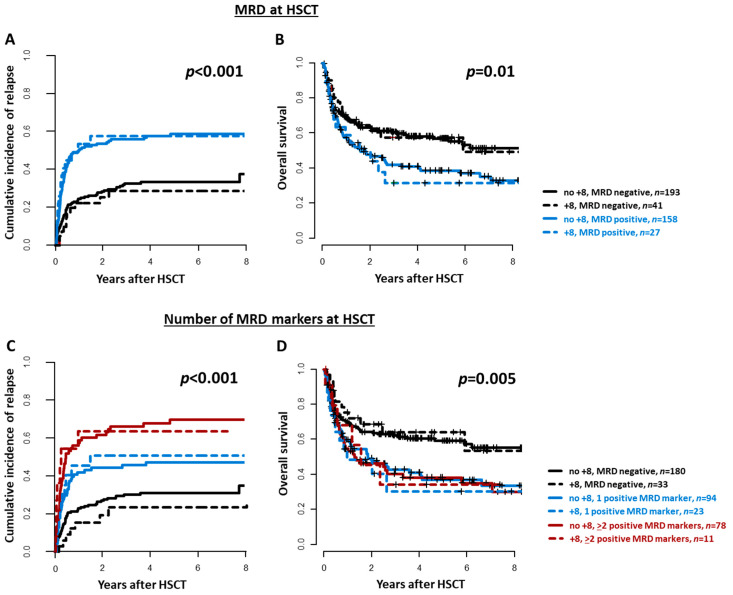
Outcomes according to the MRD status at allogeneic hematopoietic stem cell transplantation (HSCT) in AML patients with or without a trisomy 8 (*n* = 419). (**A**) Cumulative incidence of relapse (*p* for interaction = 0.64), and (**B**) Overall survival (*p* for interaction = 0.79) (MRD-positive vs. MRD-negative). (**C**) Cumulative incidence of relapse (*p* for interaction = 0.52), and (**D**) Overall survival (*p* for interaction = 0.63) according to the number of positive MRD markers (0 vs. 1 vs. 2 or more).

The MRD status according to the analyzed markers did not differ between patients with or without a trisomy 8 (Supplementary Table S4). Trisomy 8 patients with detectable MRD prior to allogeneic HSCT had a significantly higher CIR (*p* < 0.001) and significantly shorter OS (*p* = 0.004) than patients without detectable MRD. Outcomes of MRD-positive and MRD-negative trisomy 8 AML patients were comparable to those without a trisomy 8 and the corresponding MRD status at HSCT (Figure 3A,B). Moreover, trisomy 8 patients with no, one, or two or more positive MRD markers prior to HSCT had a distinguishable increasing CIR (*p* < 0.001) after allogeneic HSCT, whereas the OS was better in MRD-negative patients than in MRD-positive patients, yet the number of positive MRD markers seemed not to further impact OS (*p* for interaction = 0.63). Outcomes of patients with no, one, or two positive MRD markers were comparable between individuals with or without a trisomy 8 (Figure 3C,D).

When analyzed separately, despite limited patient numbers, each MRD marker detected in trisomy 8 patients associated with a higher CIR (mutation-based MRD *p* = 0.20; *BAALC/ABL1 p* = 0.03, *MN1/ABL1 p* = 0.002, *WT1/ABL1 p* = 0.004, FISH *p* = 0.17), resembling the relapse risk observed in AML patients without a trisomy 8 (Supplementary Figure S4).

## 4. Discussion

In the here analyzed cohort of AML patients consolidated with an allogeneic HSCT, 12% harbored a trisomy 8 AML, which is consistent with reported incidences in the literature [1,2]. Additionally, the observed characteristics of trisomy 8 patients in our study largely stand in line with previous reports. In our study, trisomy 8 patients were older [6,7], had lower white blood counts at diagnosis [6,7] and more often presented with disease of secondary origin [6]. Regarding the genetic background, we observed a lower incidence of *NPM1* mutations [6,32], no biallelic *CEBPA* mutations [6,32], but a higher incidence of *IDH1/2* [33] and *JAK2* mutations. We did not observe associations of trisomy 8 with mutated *RUNX1* or *ASXL1*, which have been previously observed in comparison to normal karyotype AML [32]. However, all karyotypes were included for comparison in our analysis, which may account for differences.

Although trisomy 8 is classified as intermediate risk AML in most risk stratification systems, initial remission rates after 7 + 3 induction therapy are reported to be around 50–70% which is slightly lower than in the average patient population with approximately 80% [7,32,34,35]. Matching these reports, in our analysis, patients with a trisomy 8 more often had to be transplanted with active disease. Nevertheless, outcomes of patients with a sole trisomy 8 seem to be comparable to those of patients with a normal karyotype [7], and in patients with one or more additional genetic aberrations are mostly driven by the accompanying chromosomal aberrations [7,13]. Adapting the established Southwest Oncology Group (SWOG) criteria for favorable, intermediate, and adverse cytogenetics, the presence of a trisomy 8 did not alter outcomes within the respective cytogenetic risk group [7]. We compared outcomes of trisomy 8 patients within the three ELN2017 genetic risk groups, which largely corresponds to the cytogenetic risk in the SWOG studies, but additionally considers molecular markers. Since we observed no distinct outcomes within the three ELN2017 risk groups for patients with or without a trisomy 8—with the caveat of low patient numbers per group—our data seconds these observations. Thus, the previously described outcome data, which was mostly derived from chemotherapy-consolidated AML patients also holds true following allogeneic HSCT and in the context of the currently used genetic risk stratification.

Regarding the optimal consolidation therapy in trisomy 8 patients, it has been suggested that chemotherapy alone may not have the ability to cure trisomy 8 AML [2,8], which was further underlined by a study by the EBMT showing more beneficial outcomes for trisomy 8 patients undergoing allogeneic HSCT compared to autologous HSCT consolidation [12]. Nevertheless, so far, no study compared outcomes of patients with or without a trisomy 8 after allogeneic HSCT. In line with previous suggestions of a potential beneficial effect of an allogeneic HSCT on outcomes of trisomy 8 patients, we did not observe higher CIR or shorter OS compared to patients without a trisomy 8, even though the incidences of intermediate or adverse ELN2017 risk was higher in trisomy 8 patients (Figure 1A,B) and trisomy 8 patients were more often transplanted with active disease.

Besides the genetic risk at diagnosis, evaluation of responses to the applied therapies during disease course is a major prognosis-defining factor in AML, which led to an effort to introduce MRD analysis into the clinical practice [36]. Despite our increasing knowledge regarding potential MRD markers in AML, we lack studies focusing on trisomy 8 AML. Until today, *NPM1* mutation-based MRD remains an established MRD method as it provides a high sensitivity for MRD detection. However, only a minority of trisomy 8 patients harbor *NPM1* mutations [6,32] (i.e., 7%, *n* = 4 in our analysis), calling for alternative MRD methods. When we adapted mutation-based MRD together with our previously established MRD markers *BAALC*, *MN1*, and *WT1* in this trisomy 8 patient cohort [17], we observed no inferior ability to detect patients at higher risk of disease reoccurrence after allogeneic HSCT compared to non-trisomy 8 patients (Supplementary Figure S4A–C). Although FISH-based MRD analysis has a limited sensitivity and does not fulfill the criteria recommended by the ELN [36], small analyses suggested a potential usefulness to predict early relapse in trisomy 8 patients remaining FISH-positive in remission [37,38]. We were able to evaluate the applicability of “FISH-MRD” at the time of HSCT in patients with material available. Although FISH-positive patients with or without a trisomy 8 had a trend for a higher CIR after allogeneic HSCT, the separation of outcome curves were not as pronounced as in the other evaluated molecular markers. However, our study surely remains restricted by lacking data on flow MRD and limited patients with data on each individual MRD markers. Subsequently, larger studies should further evaluate which MRD markers are the most useful in trisomy 8 patients in the future. Additionally, we report a long time interval and donor selection as well as supportive care regimens surely improved over the last two decades.

## 5. Conclusions

In the context of an allogeneic HSCT, the presence of a trisomy 8 alone or with additional genetic aberrations did not associate with adverse outcomes. This remained true also when the three ELN2017 groups were regarded separately. Subsequently, the presence of additional cytogenetic or molecular markers included into current risk stratification systems, rather than the presence or absence of a trisomy 8 seem to impact outcomes after HSCT. Similar to the results observed in unselected AML patient cohorts, the presence of MRD prior to allogeneic HSCT associated with a higher relapse incidence and shorter survival and allows additional risk stratification during disease course.

## Figures and Tables

**Table 1:** Clinical and genetic characteristics of AML patients undergoing allogeneic HSCT according to the presence or absence of a trisomy 8 with or without additional cytogenetic aberrations (*n* = 659).

	No Trisomy 8 Present (*n* = 578)	Trisomy 8 Present (*n* = 81)	*p*	Sole Trisomy 8 (*n* = 33)	Trisomy 8 and Additional Aberrations (*n* = 48)	*p*
**Clinical Characteristics**						
Age at diagnosis, years			0.08			0.04
median	58.7	61.9		65.4	60.4	
range	14.3–76.1	19.2–76.6		20.8–76.5	19.2–71.4	
Sex, *n* (%)			0.41			0.5
male	297 (51)	46 (57)		17 (52)	29 (60)	
female	281 (49)	35 (43)		16 (48)	19 (40)	
Disease origin, *n* (%)			0.04			0.37
secondary AML	206 (36)	39 (48)		18 (55)	21 (44)	
de novo AML	372 (64)	42 (52)		15 (45)	27 (56)	
Hemoglobin at diagnosis, g/L			0.06			0.39
median	8.7	8.9		9.5	8.8	
range	3.2–15.7	5.3–15.0		6.6–15	5.3–14.2	
Platelet count at diagnosis, × 10^9^/L			0.37			0.84
median	65	58		59	54	
range	1–950	2–305		8–305	2–218	
WBC count at diagnosis, × 10^9^/L			0.01			0.45
median	6.5	2.5		2.4	2.5	
range	0.1–385	0.6–432		0.7–432	0.6–325	
Blood blasts at diagnosis, %			0.48			0.13
median	20	22		42	19	
range	0–97	0–96		0–89	0–96	
BM blasts at diagnosis, %			0.29			0.42
median	50	50		55	44	
range	0–100	3–85		3–85	11–85	
BM CD34+/CD38− cells at diagnosis, %			0.05			0.8
median	0.7	1.5		2.3	1.3	
range	0–89	0–44.5		0–28	0–44.5	
**Genetic characteristics**						
ELN2017 genetic group, *n* (%)			0.03			<0.001
favorable	115 (26)	6 (10)		1 (6)	5 (12)	
Intermediate	146 (33)	21 (36)		13 (76)	8 (19)	
adverse	188 (42)	32 (54)		3 (18)	29 (69)	
Complex karyotype, *n* (%)			0.03			<0.001
absent	480 (86)	60 (76)		33 (100)	27 (59)	
present	77 (14)	19 (24)		0 (0)	19 (41)	
Core-Binding Factor AML, *n* (%)			1			0.14
absent	535 (95)	77 (95)		33 (100)	44 (92)	
present	29 (5)	4 (5)		0 (0)	4 (8)	
*NPM1* at diagnosis, *n* (%)			0.002			0.63
wild-type	347 (76)	54 (93)		20 (91)	34 (94)	
mutated	110 (24)	4 (7)		2 (9)	2 (6)	
*FLT3*-ITD at diagnosis, *n* (%)			0.61			0.5
absent	365 (79)	48 (83)		18 (78)	30 (86)	
present	98 (21)	10 (17)		5 (22)	5 (14)	
*CEBPA* at diagnosis, *n* (%)			0.47			1
wild-type	341 (90)	43 (86)		16 (89)	27 (84)	
mutated	40 (10)	7 (14)		2 (11)	5 (16)	
*FLT3*-TKD at diagnosis, *n* (%)			0.82			1
wild-type	387 (90)	50 (89)		18 (90)	32 (89)	
mutated	43 (10)	6 (11)		2 (10)	4 (11)	
*TET2* at diagnosis, *n* (%)			1			0.06
wild-type	84 (83)	16 (84)		5 (63)	11 (100)	
mutated	17 (17)	3 (16)		3 (37)	0 (0)	
*ASXL1* at diagnosis, *n* (%)			0.29			0.62
wild-type	106 (89)	17 (81)		6 (75)	11 (85)	
mutated	13 (11)	4 (19)		2 (25)	2 (15)	
*BCOR* at diagnosis, *n* (%)			0.73			0.04
wild-type	85 (86)	15 (83)		4 (57)	11 (100)	
mutated	14 (14)	3 (17)		3 (43)	0 (0)	
*IDH1* or *IDH2* at diagnosis, *n* (%)			0.05			0.71
wild-type	184 (78)	19 (61)		6 (55)	13 (65)	
mutated	52 (22)	12 (39)		5 (45)	7 (35)	
*JAK2* at diagnosis, *n* (%)			0.04			0.02
wild-type	118 (89)	16 (73)		4 (44)	12 (92)	
mutated	14 (11)	6 (27)		5 (56)	1 (8)	
*BAALC* copy numbers at diagnosis			0.08			0.26
median	0.06	0.19		0.06	0.37	
range	0.00–56.31	0.00–1.42		0.02–1.42	0.00–0.90	

Abbreviations: ASXL1, additional Sex Combs-Like 1 gene; BCOR, BCL6 Corepressor; BM, bone marrow; BAALC, brain and acute leukemia, cytoplasmic gene; CEBPA, CCAAT/enhancer-binding protein alpha gene; DNMT3A, DNA (cytosine-5)-methyltransferase 3A gene; ELN, European LeukemiaNet; FLT3-ITD, internal tandem duplication of the fms-like tyrosine kinase 3 gene; FLT3-TKD, tyrosine kinase mutations of the fms-like tyrosine kinase 3 gene; IDH, isocitrate dehydrogenase gene; JAK2, janus kinase 2 gene; NPM1, nucleophosmin 1 gene; TET2, Tet Methylcytosine Dioxygenase 2; WBC, white blood cell.

**Table 2:** HSCT-related characteristics of AML patients undergoing allogeneic HSCT according to the presence or absence of a trisomy 8 with or without additional cytogenetic aberrations (*n* = 659).

	No Trisomy 8 Present (*n* = 578)	Trisomy 8 Present (*n* = 81)	*p*	Sole Trisomy 8 (*n* = 33)	Trisomy 8 and Additional Aberrations (*n* = 48)	*p*
Disease status at HSCT, *n* (%)			0.04			0.09
CR/CRi1	370 (64)	56 (69)		22 (67)	34 (71)	
CR/CRi 2 or 3	102 (18)	6 (7)		5 (15)	1 (2)	
worse	106 (18)	19 (23)		6 (18)	13 (27)	
Chemotherapy cycles prior to HSCT, *n* (%)			0.79			0.43
1	177 (31)	25 (31)		12 (36)	13 (27)	
2	295 (51)	44 (54)		15 (45)	29 (60)	
≥3	105 (18)	12 (15)		6 (18)	6 (13)	
Conditioning regimens, *n* (%)			0.79			0.35
myeloablative	146 (25)	44 (54)		8 (24)	14 (29)	
reduced-intensity	96 (17)	15 (19)		4 (12)	11 (23)	
non-myeloablative	336 (58)	22 (27)		21 (64)	23 (48)	
Donor match, *n* (%)			0.73			0.33
HLA-matched related	112 (17)	14 (17)		4 (12)	10 (21)	
HLA-matched unrelated	350 (52)	47 (58)		20 (61)	27 (56)	
HLA-mismatched	100 (15)	18 (22)		7 (21)	11 (23)	
haploidentical	16 (2)	2 (2)		2 (6)	0 (0)	
Donor sex, *n* (%)			0.87			0.54
all others	490 (86)	68 (85)		27 (82)	41 (87)	
female to male	81 (14)	12 (15)		6 (18)	6 (13)	
Acute GvHD ≥ grade 2, *n* (%)			0.17			0.63
absent	373 (74)	49 (65)		19 (61)	30 (68)	
present	133 (26)	26 (35)		12 (39)	14 (32)	
Chronic GvHD, *n* (%)			0.07			0.88
absent	173 (45)	19 (36)		8 (38)	11 (34)	
limited	48 (13)	14 (36)		6 (29)	8 (25)	
extensive	160 (42)	19 (38)		7 (33)	13 (41)	

Abbreviations: CR, complete remission; CRi, complete remission with incomplete peripheral recovery; GvHD, graft versus host disease; HLA, human leukocyte antigen; HSCT, hematopoietic stem cell transplantation.

## Data Availability

Data presented in this study may be available upon request from the corresponding author.

## Supplementary Materials

The following are available online at https://www.mdpi.com/article/10.3390/cancers13225679/s1, Figure S1: ELN2017 risk distribution in AML patients with a trisomy 8 (A) without additional cytogenetic aberrations and (B) with additional cytogenetic aberrations at diagnosis; Figure S2: Outcomes of AML patients undergoing allogeneic hematopoietic stem cell transplantation (HSCT) in morphologic remission according to the presence or absence of a trisomy 8 and additional cytogenetic aberrations (sole trisomy 8 *vs* trisomy 8 and additional cytogenetic aberration *vs* others, *n* = 536). (A) Cumulative incidence of relapse/progression, and (B) Overall survival; Figure S3: Outcomes of AML patients undergoing allogeneic hematopoietic stem cell transplantation (HSCT) in first morphologic remission according to the presence or absence of a trisomy 8 and additional cytogenetic aberrations (sole trisomy 8 *vs* trisomy 8 and additional cytogenetic aberration *vs* others, *n* = 426). (A) Cumulative incidence of relapse/progression, and (B) Overall survival; Figure S4: Cumulative incidence of relapse according to the status of the included MRD markers separately at allogeneic hematopoietic stem cell transplantation (HSCT) in AML patients with or without a trisomy 8. (A) mutation-based MRD (*P* for interaction = 0.40), (B) *BAALC/ABL1*-based MRD (*P* for interaction = 0.53), (C) *MN1/ABL1*-based MRD (*P* for interaction = 0.09)*,* (D) *WT1/ABL1* (*P* for interaction = 0.17)*,* and (E) FISH-based MRD (*P* for interaction = 0.80). *P*-values reflect the comparison of all displayed curves (overall *p*-values); Table S1: Additional cytogenetic aberrations of AML patients harboring a trisomy 8 (*n* = 48); Table S2: Immunophenotype of AML patients undergoing allogeneic HSCT according to the presence or absence of a trisomy 8 with or without additional cytogenetic aberrations (*n* = 659); Table S3: Multivariate analysis; Table S4: MRD test results of the single analyzed MRD markers in AML patients undergoing allogeneic HSCT according to the presence or absence of a trisomy 8.

## References

[1] Grimwade D., Hills R.K., Moorman A.V., Walker H., Chatters S., Goldstone A.H., Wheatley K., Harrison C.J., Burnett A.K. (2010). Refinement of cytogenetic classification in acute myeloid leukemia: Determination of prognostic significance of rare recurring chromosomal abnormalities among 5876 younger adult patients treated in the United Kingdom Medical Research Council trials. Blood.

[2] Byrd J., Mrózek K., Dodge R., Carroll A. (2002). Pretreatment cytogenetic abnormalities are predictive of induction success, cumulative incidence of relapse, and overall survival in adult patients with de novo acute myeloid leukemia: Results from Cancer and Leukemia Group B (CALGB 8461). Blood.

[3] Döhner H., Estey E.H., Amadori S., Appelbaum F.R., Büchner T., Burnett A.K., Dombret H., Fenaux P., Grimwade D., Larson R.A. (2010). Diagnosis and management of acute myeloid leukemia in adults: Recommendations from an international expert panel, on behalf of the European LeukemiaNet. Blood.

[4] Döhner H., Estey E., Grimwade D., Amadori S., Appelbaum F.R., Ebert B.L., Fenaux P., Larson R.A., Levine R.L., Lo-coco F. (2017). Diagnosis and management of AML in adults: 2017 ELN recommendations from an international expert panel. Blood.

[5] Grimwade D., Walker H., Oliver F., Wheatley K., Harrison C., Harrison G., Rees J., Hann I., Stevens R., Burnett A. (1998). The importance of diagnostic cytogenetics on outcome in AML: Analysis of 1612 patients entered into the MRC AML 10 trial. Blood.

[6] Alpermann T., Haferlach C., Eder C., Nadarajah N., Meggendorfer M., Kern W., Haferlach T., Schnittger S. (2015). AML with gain of chromosome 8 as the sole chromosomal abnormality (+ 8sole) is associated with a specific molecular mutation pattern including ASXL1 mutations in 46.8% of the patients. Leuk. Res..

[7] Wolman S.R., Gundacker H., Appelbaum F.R., Slovak M.L. (2002). Impact of trisomy 8 (+8) on clinical presentation, treatment response, and survival in acute myeloid leukemia: A Southwest Oncology Group study. Blood.

[8] Schaich M., Schlenk R.F., Al-Ali H.K., Döhner H., Ganser A., Heil G., Illmer T., Krahl R., Krauter J., Sauerland C. (2007). Prognosis of acute myeloid leukemia patients up to 60 years of age exhibiting trisomy 8 within a non-complex karyotype: Individual patient data-based meta-analysis of the German Acute Myeloid Leukemia Intergroup. Haematologica.

[9] Schoch C., Haase D., Fonatsch C., Haferlach T., Löffler H., Schlegelberger B., Hossfeld D.K., Becher R., Sauerland M.C., Heinecke A. (1997). The significance of trisomy 8 in de novo acute myeloid leukaemia: The accompanying chromosome aberrations determine the prognosis. Br. J. Haematol..

[10] Konuma T., Kondo T., Yamashita T., Uchida N., Fukuda T., Ozawa Y., Ohashi K., Ogawa H., Kato C., Takahashi S. (2017). Outcome of allogeneic hematopoietic stem cell transplantation in adult patients with acute myeloid leukemia harboring trisomy 8. Ann. Hematol..

[11] Cornelissen J.J., Gratwohl A., Schlenk R.F., Sierra J., Bornhäuser M., Juliusson G., Råcil Z., Rowe J.M., Russell N., Mohty M. (2012). The European LeukemiaNet AML Working Party consensus statement on allogeneic HSCT for patients with AML in remission: An integrated-risk adapted approach. Nat. Rev. Clin. Oncol..

[12] Baron F., Labopin M., Blaise D., Gérard M.I., Forcade E., Norbert I.Y., Gorin C., Esteve J., Nagler A., Mohty M. (2021). Better leukemia-free survival with allogeneic than with autologous HCT in AML patients with isolated trisomy 8: A study from the ALWP of the EBMT. Bone Marrow Transplant..

[13] Chevallier P., Labopin M., Nagler A., Ljungman P., Verdonck L.F., Volin L., Zander A.R., Finke J., Socie G., Cordonnier C. (2009). Outcome after allogeneic transplantation for adult acute myeloid leukemia patients exhibiting isolated or associated trisomy 8 chromosomal abnormality: A survey on behalf of the ALWP of the EBMT. Bone Marrow Transplant..

[14] Thol F., Gabdoulline R., Liebich A., Klement P., Schiller J., Kandziora C., Hambach L., Stadler M., Koenecke C., Flintrop M. (2018). Measurable residual disease monitoring by ngs before allogeneic hematopoietic cell transplantation in AML. Blood.

[15] Krönke J., Schlenk R.F., Jensen K.O., Tschürtz F., Corbacioglu A., Gaidzik V.I., Paschka P., Onken S., Eiwen K., Habdank M. (2011). Monitoring of minimal residual disease in NPM1-mutated acute myeloid leukemia: A study from the German-Austrian acute myeloid leukemia study group. J. Clin. Oncol..

[16] Ivey A., Hills R.K., Simpson M.A., Jovanovic J.V., Gilkes A., Grech A., Patel Y., Bhudia N., Farah H., Mason J. (2016). Assessment of minimal residual disease in standard-risk AML. N. Engl. J. Med..

[17] Jentzsch M., Grimm J., Bill M., Brauer D., Backhaus D., Schulz J., Goldmann K., Niederwieser D., Platzbecker U., Schwind S. (2021). Prognostic relevance of remission and measurable residual disease status in AML patients prior to reduced intensity or non-myeloablative allogeneic stem cell transplantation. Blood Cancer J..

[18] Mitelman F. (1995). An International System for Human Cytogenetic Nomenclature: Recommendations of the International Standing Committee on Human Cytogenetic Nomenclature.

[19] Bill M., Jentzsch M., Grimm J., Schubert K., Lange T., Cross M., Behre G., Vucinic V., Pönisch W., Franke G.N. (2017). Prognostic impact of the European LeukemiaNet standardized reporting system in older AML patients receiving stem cell transplantation after non-myeloablative conditioning. Bone Marrow Transplant..

[20] Grimm J., Jentzsch M., Bill M., Goldmann K., Schulz J., Niederwieser D., Platzbecker U. (2020). Prognostic impact of the ELN2017 risk classi fi cation in patients with AML receiving allogeneic transplantation. Blood Adv..

[21] Jentzsch M., Bill M., Grimm J., Schulz J., Schuhmann L., Brauer D., Goldmann K., Wilke F., Franke G.-N., Behre G. (2020). High expression of the stem cell marker GPR56 at diagnosis identifies acute myeloid leukemia patients at higher relapse risk after allogeneic stem cell transplantation in context with the CD34+/CD38- population. Haematologica.

[22] Grimm J., Bill M., Jentzsch M., Beinicke S., Häntschel J., Goldmann K., Schulz J., Cross M., Franke G.-N., Behre G. (2019). Clinical impact of clonal hematopoiesis in acute myeloid leukemia patients receiving allogeneic transplantation. Bone Marrow Transplant..

[23] Metzeler K.H., Becker H., Maharry K., Radmacher M.D., Kohlschmidt J., Mrózek K., Nicolet D., Whitman S.P., Wu Y.Z., Schwind S. (2011). ASXL1 mutations identify a high-risk subgroup of older patients with primary cytogenetically normal AML within the ELN Favorable genetic category. Blood.

[24] Bill M., Grimm J., Jentzsch M., Kloss L., Goldmann K., Schulz J., Beinicke S., Häntschel J., Cross M., Vucinic V. (2018). Digital droplet PCR-based absolute quantification of pre-transplant NPM1 mutation burden predicts relapse in acute myeloid leukemia patients. Ann. Hematol..

[25] Jentzsch M., Grimm J., Bill M., Küpper J., Backhaus D., Brauer D., Schulz J., Franke G., Vucinic V., Niederwieser D. (2021). Measurable residual disease of canonical versus non-canonical *DNMT3A*, *TET2*, or *ASXL1* mutations in AML at stem cell transplantation. Bone Marrow Transplant..

[26] Bill M., Jentzsch M., Grimm J., Schmalbrock L.K., Küpper J., Backhaus D., Brauer D., Goldmann K., Franke G.-N., Vucinic V. Impact of *IDH* Mutation Detection at Diagnosis and in Remission in AML Undergoing Allogeneic Transplantation.

[27] Jentzsch M., Bill M., Grimm J., Schulz J., Goldmann K., Beinicke S., Häntschel J., Pönisch W., Franke G.-N., Vucinic V. (2017). High *BAALC* copy numbers in peripheral blood prior to allogeneic transplantation predict early relapse in acute myeloid leukemia patients. Oncotarget.

[28] Jentzsch M., Bill M., Grimm J., Schulz J., Beinicke S., Häntschel J., Goldmann K., Pönisch W., Franke G.-N., Vucinic V. (2019). Prognostic Impact of Blood *MN1* Copy Numbers Before Allogeneic Stem Cell Transplantation in Patients With Acute Myeloid Leukemia. HemaSphere.

[29] Lange T., Hubmann M., Burkhardt R., Franke G.N., Cross M., Scholz M., Leiblein S., Al-Ali H.K., Edelmann J., Thiery J. (2011). Monitoring of WT1 expression in PB and CD34 donor chimerism of BM predicts early relapse in AML and MDS patients after hematopoietic cell transplantation with reduced-intensity conditioning. Leukemia.

[30] (2017). R Development Core Team R: A Language and Environment for Statistical Computing.

[31] Gray R.J. (1988). A Class of K-Sample Tests for Comparing the Cumulative Incidence of a Competing Risk. Ann. Stat..

[32] Becker H., Maharry K., Mrozek K., Volinia S., Eisfeld A.-K., Radmacher M., Kohlschmidt J., Metzeler K., Schwind S., Whitman S. (2014). Prognostic gene mutations and distinct gene- and microRNA-expression signatures in acute myeloid leukemia with a sole trisomy 8. Leukemia.

[33] Chou W.C., Lei W.C., Ko B.S., Hou H.A., Chen C.Y., Tang J.L., Yao M., Tsay W., Wu S.J., Huang S.Y. (2011). The prognostic impact and stability of Isocitrate dehydrogenase 2 mutation in adult patients with acute myeloid leukemia. Leukemia.

[34] Döhner H., Weisdorf D.J., Bloomfield C.D. (2015). Acute myeloid leukemia. N. Engl. J. Med..

[35] Lazarevic V., Rosso A., Juliusson G., Antunovic P., Derolf Å.R., Deneberg S., Möllgård L., Uggla B., Wennström L., Wahlin A. (2017). Incidence and prognostic significance of isolated trisomies in adult acute myeloid leukemia: A population-based study from the Swedish AML Registry. Eur. J. Haematol..

[36] Schuurhuis G.J., Heuser M., Freeman S., Béne M.C., Buccisano F., Cloos J., Grimwade D., Haferlach T., Hills R.K., Hourigan C.S. (2018). Minimal/measurable residual disease in AML: A consensus document from the European LeukemiaNet MRD Working Party. Blood.

[37] Scaravaglio P., Guglielmelli T., Giugliano E., Marmont F., Audisio E., Gallo E., Saglio G., Rege-cambrin G. (2002). Detection of minimal residual disease in peripheral blood stem cells from two acute myeloid leukemia patients with trisomy 8 predicts early relapse after autologous bone marrow transplantation. Cancer Genet. Cytogenet..

[38] White D.L., Hutchins C.J., Turczynowicz S., Suttle J., Haylock D.N., Hughes T.P., Juttner C.A., To L.B. (1997). Detection of minimal residual disease in an AML patient with trisomy 8 using interphase fish. Pathology.

