# Supplementary material for: Risk Stratification, Measurable Residual Disease, and Outcomes of AML Patients with a Trisomy 8 Undergoing Allogeneic Hematopoietic Stem Cell Transplantation
Source: Cancers (Basel). 2021 Nov 13;13(22):5679. doi: 10.3390/cancers13225679 (PMC8616076; doi:10.3390/cancers13225679)
Supplement: Supplementary file 1 [file cancers-13-05679-s001.zip › cancers-1456874-supplementary.pdf]

__Supplementary Material to Backhaus *et al.*__

Risk stratification, measurable residual disease, and outcomes of AML patients with a trisomy 8 undergoing allogeneic hematopoietic stem cell transplantation

Further genetic and HSCT related information

Additional information on the surface antigen of myeloid blasts at diagnosis of acute myeloid leukemia (AML) is shown in Supplementary Table S2.

Induction therapies

The majority of AML patients received standard Cytarabine-based induction protocols, *i.e.* with conventional 7+3 (n=128), conventional 7+3 with Midostaurin (n=5), CPX-351 (n=14)[1] or sequential Azacytidine and OSHO induction (n=50); were treated within or according to the OSHO studies (#061[2] or #069,[3] under or over 60 years, n=428), the Ratify Trial (n=8),[4] the Unify Trial (ClinicalTrials.gov Identifier: NCT03512197, n=8) or the Quantum first trial (NCT02668653, n=6) and two patients were diagnosed with AML as children and treated within the AML BFM-2014 study.[5] Ten patients received Azacytidine alone.

Allogeneic HSCT in the primary patient set

Non-myeloablative (NMA) conditioning consisted of 3x30 mg/m^2 Fludarabine followed by 2 or 3 Gy total body irradiation (TBI).[6] Myeloablative conditioning (MAC) consisting of either 2x60 mg/kg body weight cyclophosphamide and 12 Gy TBI or 5x30 mg/m^2 Fludarabine and 8 Gy TBI. Reduced intensity conditioning (RIC) consisted of either busulfan (8 mg/kg orally or 6.4 mg/kg intravenously) and 5x30 mg^2 Fludarabine,[7] Fludarabine and Melphalan (n=2),[8] Fludarabine, Thiothepa, and Melphalan[9] or FLAMSA-based conditioning.[10]

For prevention of graft-versus-host disease (GvHD), all patients received an intravenous starting dose of 5 mg/kg body weight Cyclosporine A in two daily doses from day -1 which was adjusted to a whole-

blood target level of 120-150 ng/ml for patients receiving FLAMSA conditioning or 200 ng/ml for all others.

Patients undergoing NMA-HSCT additionally received Mycophenolate Mofetil 3 g per day in three daily doses in case of unrelated HSCT or 2 g per day in two daily doses in case of related HSCT. None of the patients undergoing NMA-HSCT received *in vivo* T-cell depletion.

Patients receiving FLAMSA conditioning additionally received 2 g Mycophenolate Mofetil per day, which was stopped at day 28. Patients transplanted after RIC or MAC additionally received Methotrexate 15 mg intravenously on days +1, +3, +6, and +11 after HSCT, and RIC and MAC patients transplanted from an unrelated donor additionally received *in vivo* T-cell depletion with Thymoglobulin 2 mg/kg per day for three days. Cyclosporine A was reduced starting on day +42 and stopped on day 120 following FLAMSA conditioning and for all others reduced starting on day +84 or day +180 following related or unrelated HSCT, respectively. After NMA conditioning, Mycophenolate Mofetil was stopped at day +28 following related HSCT and tapered from days +40 to +96 following unrelated HSCT. Patients were evaluated for incidence of acute GvHD and chronic GvHD using established criteria of the Glucksberg grading system.[11] Immunosuppression was prolonged or extended with systemic steroids in cases of GvHD (grade > 2 according to Glucksberg grading system).[11] Requirement for acute GvHD was engraftment while requirement for chronic GvHD was engraftment and survival for at least 100 days after HSCT.

Definition of complete remission

Complete remission (CR) was defined as the presence of <5% of blasts in bone marrow (BM), neutrophils >1.0 x 10^9/L, platelets >100 x 10^9/L, absence of blasts with Auer rods, independence of blood transfusion and no extramedullary disease.[12] CR with incomplete peripheral recovery (CRi) was defined as CR with platelets <100 x 10^9/L or neutrophils <1.0 x 10^9/L. The presence of CR or CRi was confirmed within 28 days prior to HSCT by bone marrow and peripheral blood analysis.

Multivariate Analyses

Multivariable proportional hazard models were constructed for CIR and OS to evaluate the prognostic impact of the presence of no, a sole trisomy 8 or a trisomy 8 with additional genetic aberrations in AML patients undergoing allogeneic HSCT by backward adjusting for other variables. The following variables were considered for multivariable analyses: sex, disease origin (*de novo vs* secondary), ELN2017 risk, age at HSCT (< *vs* > 60 years), disease status at HSCT (morphologic remission *vs* active disease), the MRD status at HSCT (MRD^{neg} *vs* MRD^{pos}), the HCT-CI risk score (0 *vs* 1/2 *vs* 3 or more points), performance status at HSCT (ECOG), cytomegalovirus (CMV) status of recipient and donor (high-risk [+/-] *vs* all others), donor type (matched related *vs* matched unrelated *vs* mismatched unrelated), sex of the donor (female into male *vs* all others) and the conditioning intensity (myeloablative *vs* reduced-intensity *vs* non-myeloablative). Of these, variables significant at α=.10 in univariable analyses were considered for multivariable analyses. For all endpoints, hazard/odds ratios with their corresponding 95% confidence intervals are indicated for every significant prognostic factor of the final model.

In the multivariate analysis of the whole patient cohort, the ELN2017 genetic risk at diagnosis as well as the MRD status at HSCT were significant factors for the CIR, while the ELN2017 genetic risk at diagnosis, patient age at HSCT, the presence of a morphologic remission as well as the MRD status at HSCT significantly impacted OS (Supplementary Table S3).

References

1. Lancet, J.E.; Uy, G.L.; Cortes, J.E.; Newell, L.F.; Lin, T.L.; Ritchie, E.K.; Stuart, R.K.; Strickland, S.A.; Hogge, D.; Solomon, S.R.; et al. Cpx-351 (cytarabine and daunorubicin) liposome for injection versus conventional cytarabine plus daunorubicin in older patients with newly diagnosed secondary acute myeloid leukemia. *J. Clin. Oncol.* **2018**, *36*, 2684–2692, doi:10.1200/JCO.2017.77.6112.

2. Büchner, T.; Schlenk, R.F.; Schaich, M.; Döhner, K.; Krahl, R.; Krauter, J.; Heil, G.; Krug, U.; Sauerland, M.C.; Heinecke, A.; et al. Acute Myeloid Leukemia (AML): Different treatment strategies versus a common standard arm - Combined prospective analysis by the German AML Intergroup. *J. Clin. Oncol.* **2012**, *30*, 3604–3610, doi:10.1200/JCO.2012.42.2907.

3. Niederwieser, D.; Hoffmann, V.S.; Pfirrmann, M.; Al-Ali, H.K.; Schwind, S.; Vucinic, V.; Krahl, R.; Kahl, C.; Wolf, H.-H.; Kreibich, U.; et al. Comparison of Treatment Strategies in Patients over 60 Years with AML: Final Analysis of a Prospective Randomized German AML Intergroup Study. [abstract]. In Proceedings of the Blood; 2016; Vol. 128, p. 1066.

4. Stone, R.M.; Mandrekar, S.J.; Sanford, B.L.; Laumann, K.; Geyer, S.; Bloomfield, C.D.; Thiede, C.; Prior, T.W.; Dohner, K.; Marcucci, G.; et al. Midostaurin plus chemotherapy for acute myeloid leukemia with a FLT3 Mutation. *N. Engl. J. Med.* **2017**, *377*, 454–464, doi:10.1056/NEJMoa1614359.

5. Creutzig, U.; Zimmermann, M.; Bourquin, J.P.; Dworzak, M.N.; Fleischhack, G.; Graf, N.; Klingebiel, T.; Kremens, B.; Lehrnbecher, T.; Von Neuhoff, C.; et al. Randomized trial comparing liposomal daunorubicin with idarubicin as induction for pediatric acute myeloid leukemia: Results from study AML-BFM 2004. *Blood* **2013**, *122*, 37–43, doi:10.1182/blood-2013-02-484097.

6. McSweeney, P.A.; Niederwieser, D.; Shizuru, J.A.; Sandmaier, B.M.; Molina, A.J.; Maloney, D.G.; Chauncey, T.R.; Gooley, T.A.; Hegenbart, U.; Nash, R.A.; et al. Hematopoietic cell transplantation in older patients with hematologic malignancies: Replacing high-dose cytotoxic therapy with graft-versus-tumor effects. *Blood* **2001**, *97*, 3390–3400, doi:10.1182/blood.V97.11.3390.

7. Kröger, N.; Iacobelli, S.; Franke, G.N.; Platzbecker, U.; Uddin, R.; Hübel, K.; Scheid, C.; Weber, T.; Robin, M.; Stelljes, M.; et al. Dose-reduced versus standard conditioning followed by allogeneic stem-cell transplantation for patients with myelodysplastic syndrome: A prospective randomized phase III study of the EBMT (RICMAC Trial). *J. Clin. Oncol.* **2017**, *35*, 2157–2164, doi:10.1200/JCO.2016.70.7349.

8. Baron, F.; Labopin, M.; Peniket, A.; Jindra, P.; Afanasyev, B.; Sanz, M.A.; Deconinck, E.; Nagler, A.; Mohty, M. Reduced-intensity conditioning with fludarabine and busulfan versus fludarabine and melphalan for patients with acute myeloid leukemia: A report from the Acute Leukemia Working Party of the European Group for Blood and Marrow Transplantation. *Cancer* **2015**, *121*, 1048–1055, doi:10.1002/cncr.29163.

9. Duque-Afonso, J.; Ihorst, G.; Waterhouse, M.; Zeiser, R.; Wäsch, R.; Bertz, H.; Yücel, M.; Köhler, T.; Müller-Quernheim, J.; Marks, R.; et al. Comparison of reduced-toxicity conditioning protocols using fludarabine, melphalan combined with thiotepa or carmustine in allogeneic hematopoietic cell transplantation. *Bone Marrow Transplant.* **2020**, 1–11, doi:10.1038/s41409-020-0986-2.

10. Pfrepper, C.; Klink, A.; Behre, G.; Schenk, T.; Franke, G.-N.; Jentzsch, M.; Schwind, S.; Al-Ali, H.-K.; Hochhaus, A.; Niederwieser, D.; et al. Risk factors for outcome in refractory acute myeloid leukemia patients treated with a combination of fludarabine, cytarabine, and amsacrine followed by a reduced-intensity conditioning and allogeneic stem cell transplantation. *J. Cancer Res. Clin. Oncol.* **2016**, *142*, 317–324, doi:10.1007/s00432-015-2050-y.

11. Glucksberg, H.; Storb, R.; Fefer, A.; Buckner, C.D.; Neiman, P.E.; Clift, R.A.; Lerner, K.G.; Thomas, E.D. Clinical manifestations of graft-versus-host disease in human recipients of marrow from HL-A-matched sibling donors 1974, 295–304.

12. Döhner, H.; Estey, E.H.; Amadori, S.; Appelbaum, F.R.; Büchner, T.; Burnett, A.K.; Dombret, H.; Fenaux, P.; Grimwade, D.; Larson, R.A.; et al. Diagnosis and management of acute myeloid leukemia in adults: Recommendations from an international expert panel, on behalf of the European LeukemiaNet. *Blood* **2010**, *115*, 453–474, doi:10.1182/blood-2009-07-235358.

Supplementary Tables

Supplementary Table S1. Additional cytogenetic aberrations of AML patients harboring a trisomy 8 (n=48).

UPN	Karyotype at diagnosis
70	46,XX [21]<br>46,XX,del(7)(q22q35) [4]<br>47,XX,+8 [3]
75	45,XY,der(5;17(q12;q11),ins(6;?)(q12;?),-17,del(18q) [6]<br>46,XY,idem,+8 [15]<br>45,XY,der(3;?)(q11;?),der(5;17)(q12;q11),t(6;7)(q12;?q31)del [4]
108	46,XX,t(9;11)(p22;q23) [4]<br>51,XX,+8,+8,t(9;11)(p22;q23),+12,+16,+20 [15]<br>50,XX,+8,+8,t(9;11)(p22;q23),+12,der(13;21)(q10;q10),+16,+20 [26]
128	45,XX,del(5)(q21q34),der(6)t(6;14)(p22;q23),der(8)t(8;21)(q24;q?), +8,add(13q+),-14,-21 [16]<br>46,XX [12]
180	47,XX,del(5)(q14q34),+8 [23]<br>46,XX [2]
213	51,XY,+4,+8,+9,+19,+21 [12]<br>46,XY [13]
251	47,XX,+8.ish inv(16)(p13q22)(pcp16q sp) [19]<br>46,XX [5]
261	48,XY,del(5)(q31q34),+8,+21 [13]<br>46,XY [12]
269	48,XY,+8,+13 [20]<br>46,XY [10]
287	47,XY,del(7)(q21q36),+8 [15]<br>46,XY [10]
301	47,XX,+8,inv(16)(p13q22) [7]<br>48,XX,idem,+21 [18]; 46,XX [4]
316	47,XX,+8,t(11;19)(q23;p13) [19]<br>46,XX [6]
384	46,X,-Y,t(1;8;21)(p13;q22;q22),+8 [30]
412	48,XY,+8,+21[3]<br>46,XY[27]

417	46,XX,der(1)t(1;16)(?;?),der(1;7)(q10;p10),del(3)(q13),der(6)t(6;16)(p21;?q10),+8,-16[19]<br>47,XX,der(1;7)(q10;p10),+8[10]<br>48,XX,der(1;7)(q10;p10),+8,+8[2]
423	47,XY,+8,iso(17)(q10) [11]<br>46,XY,iso(17)(q10) [6]
439	47,XY,t(3;11)(p21;q23),+8 [28]<br>46,XY [2]
469	52-53,XY,+Y,del(5)(q12q34),+6,del(7)(q?21q35),+8,+9,+19,+1-2 mar [5]<br>46,XY [8]
481	48,XY,+8,+11 [27]
498	47,XX,+8 [16]<br>47,XX,+11 [5]<br>46,XX [14]
522	46,XY,del(20q)[16]<br>47,XY,+8,del(20q)[5]<br>47,XY,+8,del(13)(q14q21),del(20q)[9]<br>46,XY[2]
532	46,XX,del(7)(q21q36),del(20q)[4]<br>47,XX,del(7)(q21q36),+8,del(20q)[21]
536	47,XY,+8,t(9;11)(p21;q23)[4]<br>46,XY [20]
554	47,XX,t(6;21)(q15;q21),+der(6)t(6;21)(q15;q21) [23]<br>47,XX,t(6;21)(q15;q21),+8 [5]<br>46,XX [3]
563	43-44,XX,-3,der(5;17)(p10;q10),-7,+8[cp12]<br>46,XX[10]
573	50,XY,+4+8+13+22[3]<br>46XY[22]
581	49,XY,t(2;4;17;?)(p?;q?26;?;?),+8,+8,der(17)(?),der(21)(?),+mar[19]<br>46,XY [1]
584	47,XY,+8 [28]<br>47,XY,+8,del(20)(q12) [2]
622	43-46,XY,del(5)(q14q34),-7,+8,dic(12;13)(p12;p11),der(15;16)(q10;p10),add(17)(p13),+0-3mar[cp22]<br>46,XY[8]
652	47,XY,+8,ins(10;11)(p?;q?23q?23)[21]<br>46,XY[4]

657	48,XY,+8,+22[22]<br>46,XX [3]
701	46,XX [9]; 47,XX,+8 [6]<br>45,XX,-7 [4]
703	45,XY,der(1;7)(q10;p10) [22]<br>46,idem,+8 [6]<br>45,X,-Y [7]
736	54-55,XX,+der(1)t(1;?)(q11;?),+2,del(5)(q14q34),+del(5)(q14q34),+8,+11,add(12)(p13), +13,+der(14)t(14;17)(q32;q11),-17,+21,+22,+mar[cp11]<br>54-55,XX,+der(1)t(1;?)(q11;?),+2, del(5)(q14q34),+del(5)(q14q34),+8,+11,add(12)(p13),+14,+21,+22,+mar[cp11]
744	47,XY,+8,inv(16)(q22p13) [16]<br>46,XY [9]
765	47,XY,+8[8]/48,XY,+8,+21 [6]<br>46,XY [12]
772	47,XY,+8[13]/ 48,XY,+8,+11 [8]<br>46,XY [4]
782	46,XY,der(9)del(9)(q22q33)ins(9)(q?21p21p2?2),ins(10;11)(p1?2;q23q13),del(11)(q12q23) [8]<br>53,idem,+4,+6,+8,+der(9)del(9)(q22q33)ins(9)(q?21p21p2?2),+13,+20,+21 [3]<br>46,XY [7]
788	47,XX,+8 [17]<br>45,XX,der(3)t(3;17)(q2?8;q?),der(17;20)(q10;p10) [5]<br>46,XX [9]
791	45,XY,-7,t(3;10)(p14;q24),t(16;21)(p11;q22) [20]<br>46,idem,+8 [1] .ish+8(cen8x3,MYCx3) [3]
803	47,XY,+8,t(13;17)(q14;q22) [12]<br>46,XY [18]
817	43,X,der(X)t(X;?)(p11;?),add(1q+),del(3)(q11q18),-4,del(5)(q13q34),der(7;8)(q11;q11),+8,der(8)t(8;?)(p11;?),der(9)t(?1;9)(p34;q?31),add(10p+),del(11)(p14p15), der(11;16)(q11;q11),add(12p+),add(12q+),der(13)(?),-16,add(16q+),-17,-18,add(18q+), der(21;?)(q11;?),der(22;?)(q11;?),+2mar [cp10]<br>44,XX,del(5)(q13q34),der(7;17)(q11;q11),der(14;15)(q11;q11),der(16;?) (q?;?),-18,add(18p+),der(21;?)(q11;?),-22,+dmin [cp8]<br>43,XX,der(X;?17)(q11;q?11),der(2)t(2;9)(q1?3;q?22),der(3)?inv(3) (q?13q?27),del(5)(q13q34),der(7)t(7;15)(q32;q21),+8,der(?8)(8qtel->8q11::cen::10q11->10q2?4::?),der(15)del(15q21q2?6),-16,-17,-18[cp4]<br>44,XX,del(5)(q13q34),der(7;17)(q11;q11),der(12;14)(q11;q11),der(18;?)(q11;?),-19,-21,der(?22)t(22;?)(q11;?)[cp4]<br>40~43,XX,add(2p+),der(4)t(4;?)(q2?6;?),del(5)(q13q34),der(7;8)(q11;q11),-8,add(8p+),del(9)(q2?2q3?4),-10, add(10q+),add(11q+),12,add(12p+), der(12;?16)(q11;p11),-14,-16,-17,der(17;19)(q11;q11),-18,-22,der(22)t(22;?)(q12;?), add(22p+),+mar[cp3]<br>42~43,XX,del(2)(q22q?35),der(4)t(4;?)(q2?6;?),+9,der(9;?)(p23;?),del(9)(q13q33),add(10q+),del(13)(q13q34),add(15q+),der(16;?)(p11;?),-16,-17,-18,del(18)(q21q23),der(19)t(11;19)(q14;p11),-22,der(?22)t(22;?)(q11;?),+mar [cp3]<br>41,X,-X,der(2)del(2)(q22q?35)t(2;8)(q21;q23),der(3)?inv(3)(q?13q?27),del(5)(q13q34),del(6)(q2?3q2?6),t(7;12)(q21; q24),der(?8)(8qtel->8q11::cen::10q11->10q2?4::?),-10,add(16q+),der(?17)del(17)(p13),-18,-20,-21,add(22)(p+)[cp2]

	43,XX, add(2p+),der(4)t(4;?)(q2?6;?),del(5)(q13q34),der(6)t(6;8)(p25;q21),-8,add(8p+),add(10q+),-12,add(16q+),-17,-18,+mar [2]<br>46,XX [2]
821	45,XX,-5,?t(6;21)(p21;q22),del(12)(p13),-18,+mar [4]<br>46,idem,+8[2]<br>45,idem,+8,-11 [2]<br>48,idem,+8,der(11)(?),+2mar,1-2dmin [4]
860	58,XY,+1,+2,+6,+8,+10,+11,+14,+16,+19,+21,+21,+22 [cp2]<br>46,XY [30]
866	47,XX,der(1;7)(q10;p10),+8 [25]<br>46,XX [5]
872	47,XX,+8 [2] .ish+8(RUNX1T1x3) [2]<br>46,XX [29]
880	44-45,XY,add(2)(q2?6),-3,del(5)(q21q34),del(6)(q16q23),inv(7)(p15q35),+8,der(12)t(3;12)(?;q24),add(15)(q?21),add(17)(p12),add(21)(q2?1),-22 [4]<br>54,XY,+1,+4,del(5)(q21q34),+6,del(6)(q16q23),inv(7)(p15q35),+8,+11,+14,+21,+22[6]

Supplementary Table S2. Immunophenotype of AML patients undergoing allogeneic HSCT according to the presence or absence of a trisomy 8 with or without additional cytogenetic aberrations (n=659).

	no trisomy 8 present (n=578)	trisomy 8 present (n=81)	*P*	sole trisomy 8 (n=33)	trisomy 8 and additional aberrations (n=48)	*P*
BM CD34 expression, %			.03			.95
Median	24	35		27	38	
range	0-97	0.2-90		0.7-79	0.2-90	
BM CD38 expression, %			.06			.55
Median	72	65		65	64	
Range	0.5-98	20-97		20-89	34-97	
BM CD117 expression, %			.80			.40
Median	34	35		39	33	
Range	0-96	0.5-93		2.4-82	0.5-93	
BM CD7 expression, %			.74			.16
Median	17	17		17	20	
Range	1-96	3-69		3-39	3-69	
BM CD56 expression, %			.56			.19
Median	16.5	14		8	14	
Range	0.5-93	1-91		2-69	2-87	
BM Glykophorin A expression, %			.003			.35
Median	10	15		16	15	
Range	0-90	1-55		3-52	1-55	
BM CD2 expression, %			.14			.40
Median	15	16		13	18	
range	1-97	5-55		5-45	6-55	
BM CD11b expression, %			.21			.03
Median	16	11		7	13	
range	0.5-97	1-92		1-41	2-92	
BM CD13 expression, %			.33			.79
Median	57	52		51	54	
range	0.5-97	7-94		7-94	9-86	
BM CD33 expression, %			.01			.008
Median	64	50		33	59	
range	1-98	8-94		8-90	16-94	

BM CD15 expression, %			.19			.02
Median	28	21		14	30	
range	1-97	4-91		5-55	4-91	
BM CD65 expression, %			.74			.003
Median	17	14		9	24	
Range	0.5-93	1-91		1-53	6-91	
BM CD14 expression, %			.84			.76
Median	2	3		3	3	
Range	0.5-74	0.5-35		0.5-35	0.5-26	
BM CD64 expression, %			.46			.05
Median	15	13		9	15	
range	0-98	1-91		1-47	1-91	
BM CD45 expression, %			.002			.30
Median	92	86		82	87	
Range	6-100	29-99		40-99	29-99	
BM CD61 expression, %			.08			.45
Median	5	8		5	8	
Range	0.5-72	0.5-34		0.5-22	0.5-34	

Abbreviations: BM, bone marrow; CD, cluster of differentiation.

Supplementary Table S3. Multivariate analysis

	Cumulative incidence of relapse/progression		Overall survival	
	HR* (95% CI)	*P*	OR** (95% CI)	*P*
**ELN2017 genetic risk (adverse *vs* intermediate *vs* favorable)**	1.82 (1.46-2.27)	<.001	0.63 (0.51-0.78)	<.001
**Age at HSCT (> *vs* < 60 years)**	-	-	0.55 (0.40-0.75)	<.001
**Morphologic remission at HSCT (absent *vs* present)**	-	-	0.56 (0.36-0.86)	.008
**Pre-HSCT remission status (MRD^{pos} *vs* MRD^{neg})**	2.44 (1.75-3.39)	<.001	0.68 (0.49-0.94)	.02

Abbreviations: CI, confidence interval; ELN2017, European LeukemiaNet 2017; HSCT, hematopoietic stem cell transplantation; MRD, measurable residual disease.

***HR, hazard ratio, <1 (>1) indicate lower (higher) risk of relapse for the first category listed for the dichotomous variables for the lower (higher) values of the continuous variables.**
****OR, odds ratio, <1 (>1) indicate lower (higher) chance of survival for the first category listed for the dichotomous variables.**

Variables considered in the models were those significant at α=0.10 in univariate analyses. For CIR endpoint, variables considered were: ELN2017 genetic risk group, conditioning regimen (NMA *vs* RIC *vs* MAC), morphologic remission status at HSCT (absent *vs* present), pre-HSCT remission status (MRD^{pos} *vs* MRD^{neg}), and age at HSCT. For OS endpoint, variables considered were: disease origin (secondary or therapy-related *vs de novo*), ELN2017 genetic risk group, donor type (matched related *vs* matched unrelated *vs* mismatched unrelated, conditioning regimen (NMA *vs* RIC *vs* MAC), morphologic remission status at HSCT (absent *vs* present), pre-HSCT remission status (MRD^{pos} *vs* MRD^{neg}), and age at HSCT.

Supplementary Table S4. MRD test results of the single analyzed MRD markers in AML patients undergoing allogeneic HSCT according to the presence or absence of a trisomy 8.

	**no trisomy 8 present (n=578)**	**trisomy 8 present (n=81)**	***P***
Mutation-based MRD, n (%)			1
negative	52 (44)	9 (64)	
positive	67 (56)	5 (36)	
BAALC/ABL1 expression MRD, n (%)			.33
negative	167 (71)	22 (63)	
positive	68 (29)	13 (37)	
MN1/ABL1 expression MRD, n (%)			.83
negative	184 (77)	27 (75)	
positive	54 (23)	9 (25)	
WT1/ABL1 expression MRD, n (%)			.28
negative	129 (75)	21 (66)	
positive	43 (25)	11 (34)	
FISH MRD, n (%)			.39
negative	106 (68)	31 (61)	
positive	50 (32)	20 (39)	

Abbreviations: *BAALC*, brain and acute leukemia, cytoplasmic; FISH, fluorescence in-sito hybridization; *MN1*, meningeoma-1; MRD, measurable residual disease; *WT1*, wilm's tumor gene 1.

Supplementary Figures

Supplementary Figure S1

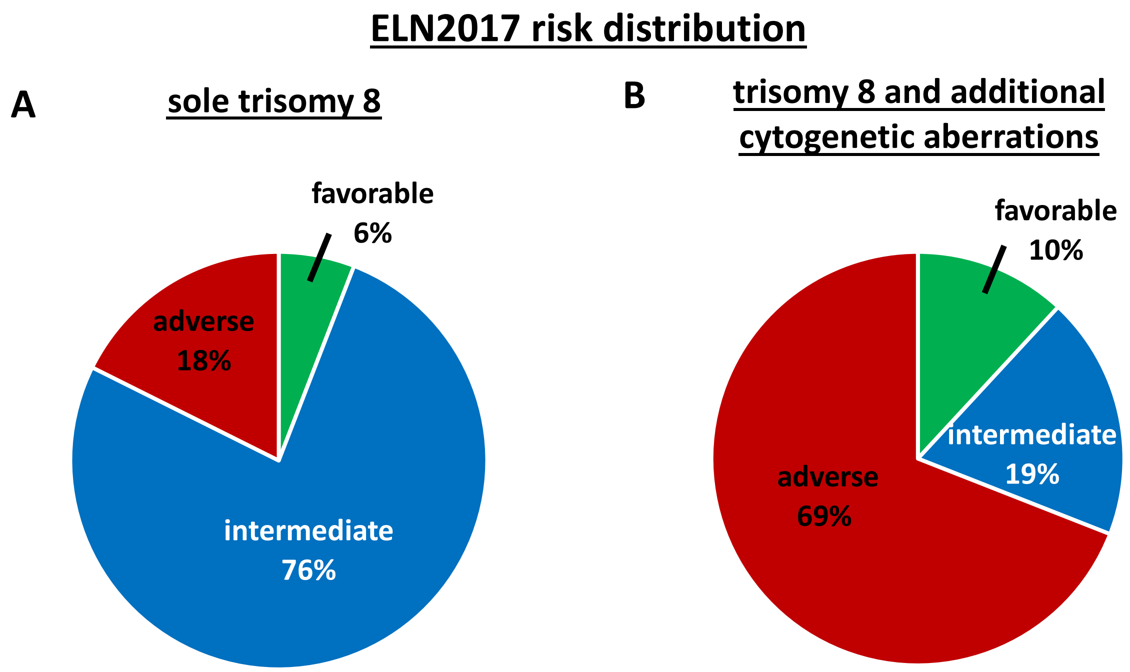

Supplementary Figure S1. ELN2017 risk distribution in AML patients with a trisomy 8 (A) without additional cytogenetic aberrations and **(B)** with additional cytogenetic aberrations at diagnosis.

Supplementary Figure S2

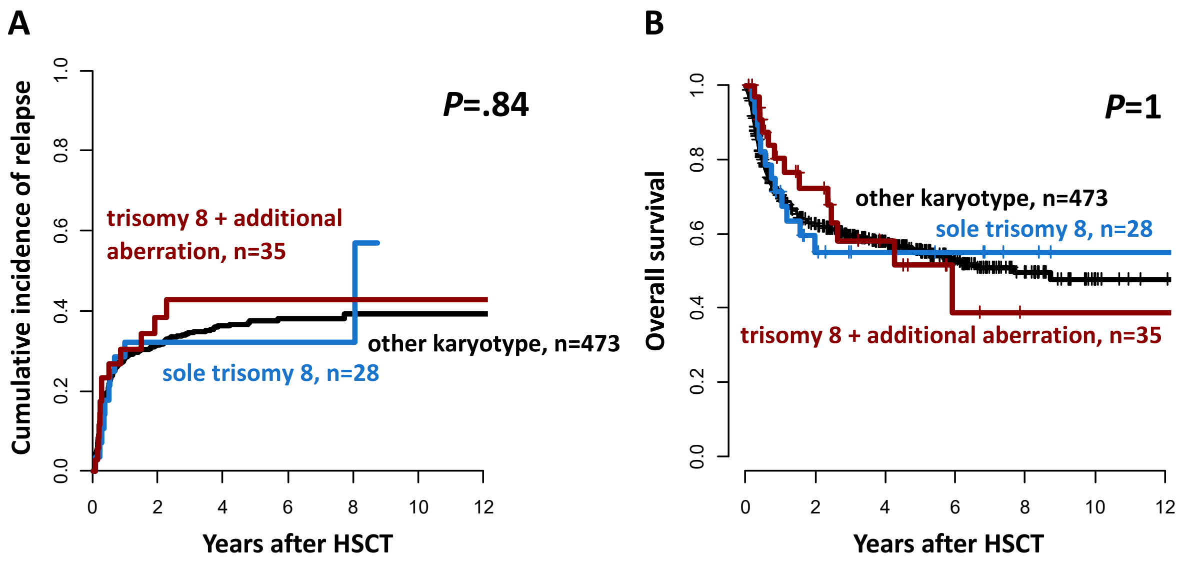

Supplementary Figure S2. Outcomes of AML patients undergoing allogeneic hematopoietic stem cell transplantation (HSCT) in morphologic remission according to the presence or absence of a trisomy 8 and additional cytogenetic aberrations (sole trisomy 8 *vs* trisomy 8 and additional cytogenetic aberration *vs* others, n=536). (A) Cumulative incidence of relapse/progression, and **(B)** Overall survival.

Supplementary Figure S3

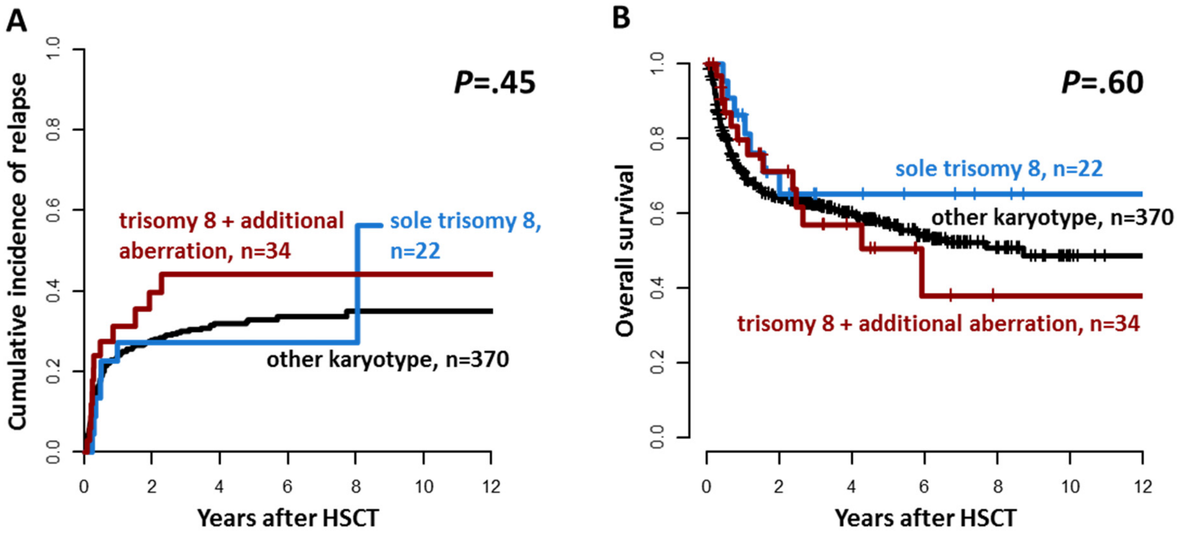

Supplementary Figure S3. Outcomes of AML patients undergoing allogeneic hematopoietic stem cell transplantation (HSCT) in first morphologic remission according to the presence or absence of a trisomy 8 and additional cytogenetic aberrations (sole trisomy 8 *vs* trisomy 8 and additional cytogenetic aberration *vs* others, n=426). (A) Cumulative incidence of relapse/progression, and **(B)** Overall survival.

Supplementary Figure S4

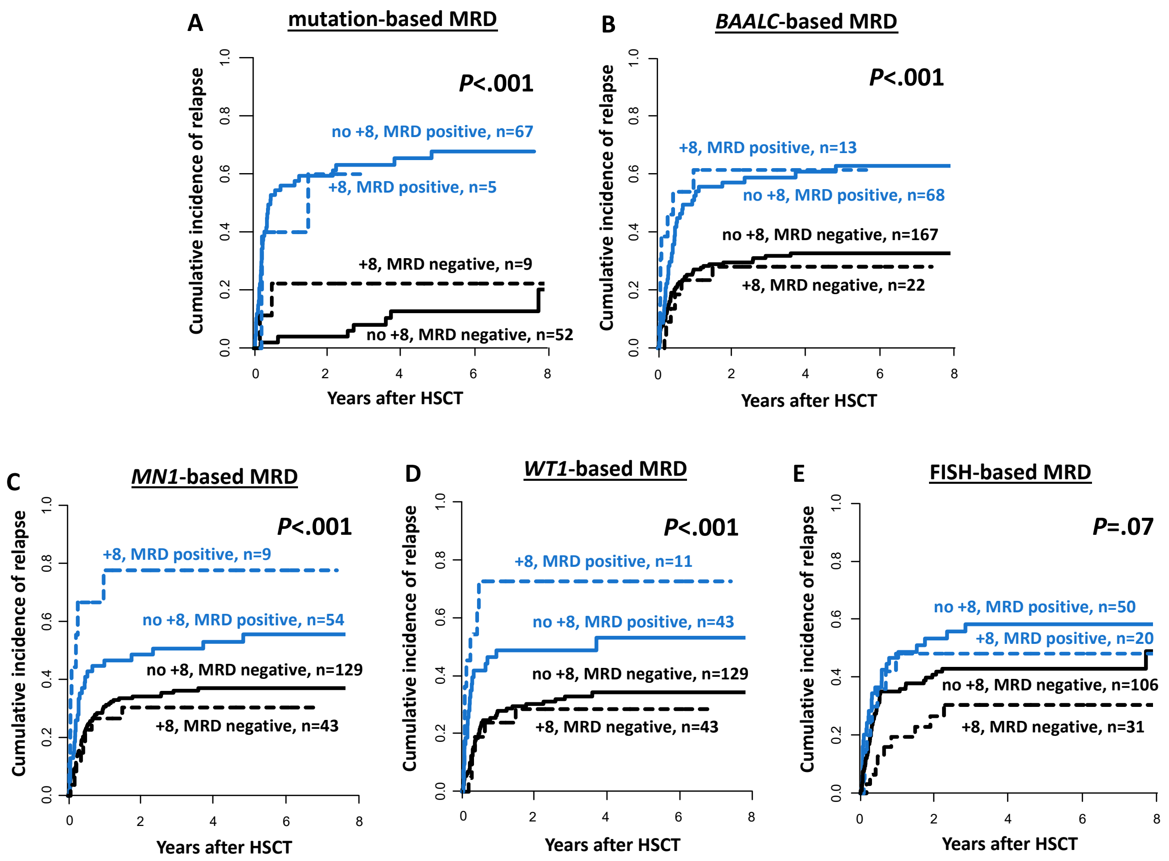

Supplementary Figure S4. Cumulative incidence of relapse according to the status of the included MRD markers separately at allogeneic hematopoietic stem cell transplantation (HSCT) in AML patients with or without a trisomy 8. (A) mutation-based MRD (*P* for interaction =.40), **(B)** *BAALC/ABL1*-based MRD (*P* for interaction =.53), **(C)** *MN1/ABL1*-based MRD (*P* for interaction =.09), **(D)** *WT1/ABL1* (*P* for interaction =.17), and **(E)** FISH-based MRD (*P* for interaction =.80).

P-values reflect the comparison of all displayed curves (overall *P*-values).
